# Generation Mechanism and Reynolds Number Regulation of Multi-Peak Oscillatory Concentration Gradients in Multi-Layer Vertical-Stepped Microchannels

**DOI:** 10.3390/mi17030294

**Published:** 2026-02-27

**Authors:** Zengliang Hu, Minghai Li, Guangda Liu, Xiaohui Jia, Zhenyu Fan

**Affiliations:** 1 School of Mechanical Engineering, Dalian Jiaotong University, Dalian 116028, China; 2 School of Chemical Engineering and Machinery, Liaodong University, Dandong 118001, China; 3 Zhan Tianyou College, Dalian Jiaotong University, Dalian 116028, China; 4 School of Innovation and Entrepreneurship Education, Liaodong University, Dandong 118001, China; 5 School of Continuing Education, Liaodong University, Dandong 118001, China; 6 Xinhua Standard Parts Factory Co., Ltd., Shenyang 110141, China

**Keywords:** microfluidic chip, concentration gradient generator, multi-layer microchannel structure, finite element analysis, oscillatory concentration gradient

## Abstract

This study systematically investigates the flow characteristics, mixing efficiency, and concentration gradient generation (CGG) capabilities of three types of vertical-stepped main-channel microfluidic concentration gradient generators—the upward vertical-step (UVS-GG), downward vertical-step (DVS-GG), and straight horizontal channel (SHC-GG)—under different Reynolds numbers (Re) through numerical simulation and comparative analysis. Using numerical simulations, the research reveals the universal transition of flow regimes from diffusion-dominated to convection-dominated and reports the emergence of a “multi-peak oscillatory concentration gradient” phenomenon under stepped geometries and high Re (Re = 100, 200). The results indicate that the SHC-GG can generate monotonic gradients at low Re, making it an ideal baseline configuration. In contrast, UVS-GG and DVS-GG enhance mixing and enable the programming of complex concentration distributions through unique inertia–geometry coupling effects. The synergistic interaction between geometric configuration and Re is identified as the core mechanism for regulating concentration field morphology and device performance. This study provides key theoretical and design foundations for the rational design of microfluidic gradient generators targeting applications such as biological screening, chemical analysis, and material synthesis.

## 1. Introduction

As one of the most promising interdisciplinary frontiers in the 21st century, microfluidic technology has demonstrated revolutionary potential in chemical analysis, biomedicine, drug screening, and tissue engineering [1]. By precisely manipulating minute fluid volumes within micron-scale channels, this technology achieves high throughput, high sensitivity, and low reagent consumption—advantages difficult to attain with traditional macroscopic methods [2]. Among various microfluidic research directions, concentration gradient generators (CGGs) have become a hot topic in recent years due to their unique functionality and wide applicability. Concentration gradients are ubiquitous and critical physicochemical factors in biological systems, playing significant roles in physiological and pathological processes such as cell chemotaxis, drug action, embryonic development, and tumor metastasis [3,4,5].

Traditional methods for generating concentration gradients suffer from limitations such as gradient instability and long processing times. Leveraging precise spatiotemporal control, microfluidic CGGs can produce stable, reproducible, and complex concentration distributions, providing a powerful tool platform for related research. Based on the gradient generation principle, CGGs can be mainly categorized into static diffusion-based and flow-based types. Designs relying solely on static diffusion form gradients only through diffusion, making them difficult to achieve spatiotemporal stability, often featuring complex structures and challenging fabrication. In contrast, flow-based designs achieve efficient and stable gradient control through the synergy of convection and diffusion, often with simpler single-layer structures [6,7]. Classic models based on flow-type CGGs include diffusion-based, pressure-balanced, T-junction, hydrogel-based, and tree-shaped CGGs [8,9]. Numerous CGG designs have been widely used in various biological, drug screening, and chemical studies [10,11,12,13]. Rismanian et al. designed a CGG capable of generating continuous concentration gradients of multiple reagents in millimeter-scale samples. This device can generate continuous concentration gradients for two reagents and deliver combined concentrations to millimeter-sized samples [14]. Zhang et al. employed machine learning techniques and interpolation algorithms to enable real-time analysis of the current concentration distribution in gradient generators with different inlet configurations, realizing the potential for automated design and computer-aided design in microfluidics [15]. Dai et al. designed a microfluidic device capable of cyclically generating different concentration gradient flows and droplets for preparing paclitaxel concentration gradients, and found that the survival rate of 4T1 cells was negatively correlated with drug concentration [16]. Among these studies, the classic tree-shaped model is the most widely applied. Liu et al. integrated a tree-shaped CGG with a microfluidic single-cell trapping array, constructing a platform that enables spatiotemporally precise generation of nanoparticle concentrations and real-time monitoring of single-cell behavior via a high-content system. This platform can exclude population averaging effects, allowing precise characterization of cellular responses to gradient concentrations at the single-cell level [17]. Wang et al. developed a microfluidic platform based on a tree-shaped CGG to assess tumor cell heterogeneity and drug resistance. The platform demonstrated that the combination of imatinib and resveratrol showed significantly better inhibitory effects on single cells and clones compared to single drugs, with clone cells exhibiting stronger drug resistance [18].

As an important development direction in microfluidic technology, multi-layer CGGs achieve higher functional complexity integration and more flexible system construction within limited planar areas through three-dimensional structural design and integration. Yoon et al. developed a multi-layer CGG cell-culture chamber platform that enabled simultaneous generation and integrated manipulation of linear and logarithmic concentration gradients, which was successfully applied to combination chemotherapy drug screening for bladder cancer cells [19]. Tang et al. proposed a highly automated linear CGG based on a multi-layer centrifugal microfluidic platform. This device successfully determined the minimum inhibitory concentration of ampicillin against Escherichia coli [20].

The finite element simulation method offers significant advantages in the development of CGGs, primarily manifested in its ability to perform multi-physics coupled simulations of complex microfluidic networks, thereby enabling structural optimization and performance prediction during the device design stage [21]. By solving the Navier–Stokes equations and the convection–diffusion equation, simulations can accurately model fluid flow and mass transport processes at the micro-scale, revealing the influence of parameters such as flow rate ratio and channel geometry on gradient patterns, providing reliable theoretical guidance for experiments [22]. Furthermore, the finite element method supports dynamic process analysis, capturing the time-dependent evolution of gradient formation, making it suitable for studying cellular responses in dynamic chemical environments [23]. Combining simulation and experimental results not only validates model accuracy but also provides an in-depth interpretation of the physical mechanisms behind gradient generation, promoting device development towards integration and functionalization [24,25]. With parametric design and optimization algorithms, finite element simulation further facilitates the standardization and automated development of CGGs, offering robust technical support for their widespread application in drug screening, cell chemotaxis research, and high-throughput bioassays [26]. It is noteworthy that, based on the emerging field of inertial microfluidics, the ingenious design of microchannel geometry can modulate inertial effects to induce enhanced secondary flow and vortex structures, thereby significantly improving mixing and transport efficiency [27]. Ghazimirsaeed et al. employed numerical simulations to investigate the effects of different cross-sectional shapes on droplet flow within serpentine microchannels, and further examined the secondary flows, inertial effects, and chemical reactions inside the droplets [28]. Shi et al. conducted a numerical study on how lateral structures generate secondary flow in laminar flows and established that a velocity difference between the lateral structure and the main channel is the necessary condition for Dean vortex formation [29]. Thus, combining three-dimensional structural design with secondary flow offers new insights for developing high-performance, functionally integrated concentration gradient generators.

Based on the above review, this paper, integrating tree-shaped CGGs, multi-layer microfluidic chips, and finite element simulation, designed three tree-shaped CGGs with different main-channel stepped structures. Using numerical simulations, the stepped structure developed in this study may enable a new approach for generating non-uniform oscillatory gradients, differing from conventional tree-shaped CGGs based on flow-splitting or diffusion principles. In the study, seven different Reynolds numbers were selected to comparatively analyze fluid state changes based on factors such as inter-layer variations within the same structure, same-layer comparisons across different structures, and pressure drops between inlets and outlets. Concentration gradient curves at the outlet under different Re values were obtained, leading to the acquisition of multi-peak oscillatory CGGs. It should be noted that these insights are primarily derived from numerical observations, which highlight the need for future experimental validation.

## 2. Structural Design of the Three Types of Concentration Gradient Generators

**Figure 1:**
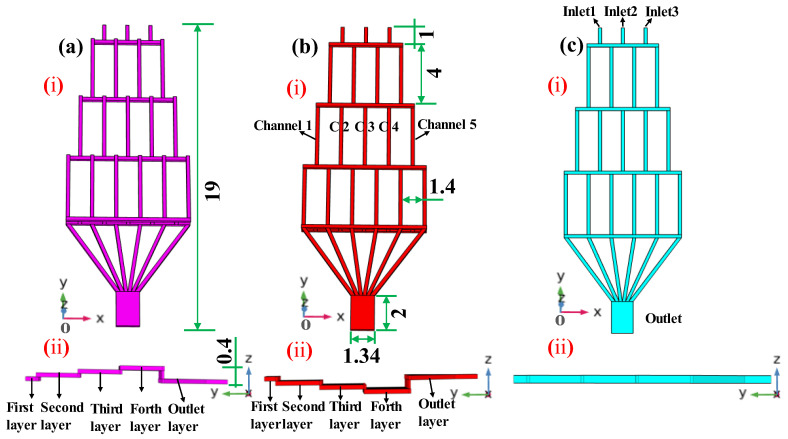
Structural schematic diagrams of the three proposed microfluidic devices (unit: mm). (**a**) UVS-GG device, (**b**) DVS-GG device, (**c**) SHC-GG device, (i) view of the main structure, (ii) view of the main structure in the *y*-*z* plane.

This experiment designed three types of CGGs with different structures: the Upward vertical-step main-channel CGG (UVS-GG), the downward vertical-step main-channel CGG (DVS-GG), and the straight horizontal main-channel CGG (SHC-GG). All three devices feature left–right symmetrical microchannel structures and share three identical independent inlet structures and parallel downstream outlet arrays, as shown in Figure 1. Considering the influence of multi-layer microchannel paths on the internal flow field and mass transport, the core geometric difference among the three lies in the four-layer hierarchical morphology of the intermediate main mixing channel connecting the inlets and outlets. For each type, the inlet and outlet lengths were 1 mm and 2 mm respectively, with a mixing microchannel width of 0.2 mm. To enhance final solution mixing while maintaining the slanted channel width of 0.2 mm, the outlet width was designed as 1.34 mm. The overall microstructure had a length of 19 mm, a width of 5 mm, and a vertical step height of 0.2 mm.

Figure 1(ai) show the main structure of the UVS-GG. Figure 1(aii) illustrate its main-channel-layered structure, which adopted a “climbing–falling back” composite geometric configuration. Starting from the inlet plane, a series of short horizontal platforms with vertical ascent angles climbed upward step by step (four steps in total). Near the outlet region, it rapidly descended, falling back in one large vertical drop to the outlet plane. This structure primarily measured complex flow field changes induced by sharp ascent and descent. Figure 1(bi) show the main structure of the DVS-GG. Figure 1(bii) present its “settling–lifting back” main channel configuration. After the inlet, the main channel immediately descended stepwise through multiple vertical levels to a lower plane, followed by a sharp vertical rise segment that quickly lifted it back to the outlet plane. This structure aided in studying the deposition and distribution effects of gravity or centrifugal force on particles or heavier components. The two different structures shared the design feature of coplanar inlet and outlet layers. The SHC-GG is a single-layer CGG structure, as shown in Figure 1c. Its inlet, main channel, and outlet lay on the same horizontal plane. This structure entirely eliminated the additional acceleration field and flow direction changes introduced by vertical geometric variations, ensuring the flow remains in a stable pressure-driven laminar state. To better describe the location of the microchannels within each layer, the naming convention Channel N (abbreviated as C *n*, where *n* = 1, 2, …) was used, which designated the microchannels in the order of their increasing *x*-coordinates (left to right).

## 3. Numerical Simulation Governing Equations

This simulation study was conducted based on the COMSOL Multiphysics 6.2 finite element simulation platform. The finite element method is currently the most mainstream numerical method in simulation research, and the physical governing equations are the foundation for achieving numerical simulation. In this study, the core governing equations mainly included the Navier–Stokes equations (N-S equations) in the following forms:

(1) $$\rho \frac{\partial \nu }{\partial t}+\rho \left(\nu \cdot \nabla \right)\nu +\nabla p-\eta \nabla^{2}\nu =0$$

(2) ∇⋅ν=0

where *v* represents the velocity vector; *ρ* is the fluid density; *η* is the dynamic viscosity; and *p* is the pressure. Equation (1) describes the conservation of momentum, while Equation (2) represents the conservation of mass for an incompressible fluid.

Furthermore, the convection–diffusion equation was also one of the key governing equations in this simulation, expressed as:

(3) $$\frac{\partial c}{\partial t}+\nabla \cdot \left(-D\nabla c\right)=-u\cdot \nabla c$$

where *c* is the substance concentration; *D* is the diffusion coefficient; and u remains the fluid velocity vector. This equation is used to describe the transport behavior of solute in the flow field.

The dimensionless Reynolds numbers (Re) for fluid flow were the main parameter of this study. They can describe the properties of fluid flow under different conditions by measuring the ratio of fluid inertia to viscous forces, expressed as:

(4) Re=ρωdμ

where *d* represents the characteristic length, equivalent to the hydraulic diameter for non-circular cross-sections; *µ* denotes the kinematic viscosity coefficient; *ω* represents the fluid flow velocity; and *ρ* is the fluid density. As can be seen from the formula, the Re was directly proportional to the flow velocity and inversely proportional to the kinematic viscosity coefficient.

Since the inlet concentrations were fixed at 0 mol/L (side inlets) and 1 mol/L (central inlet), the maximum concentration variance at the outlet cross-section occurred when the fluid was completely segregated into pure 0 and pure 1 streams. Under this condition, the theoretical maximum variance was given by:

(5) $$\sigma_{\max}^{2}=\bar{c}(1-\bar{c})$$

where $\bar{c}$ is the mean concentration at the outlet plane. The mixing efficiency was then defined as:

(6) $$\theta =1-\frac{\sigma^{2}}{\sigma_{\max}^{2}}=1-\frac{\sigma^{2}}{\bar{c}(1-\bar{c})}$$

This formulation ensures that *θ* = 0 for a completely unmixed state and *θ* = 1 for perfect mixing.

The parameters for the three types of devices in this simulation were consistent. According to the simulation requirements, the study was conducted in three-dimensional space, primarily examining the laminar steady-state flow process of fluid within the microchannels. The fluid was an aqueous solution with a constant diffusion coefficient of 1 × 10^−10^ m^2^/s. To ensure numerical stability in the convection-dominated regime, consistent stabilization methods were employed in both the Laminar Flow and Transport of Diluted Species interfaces. Specifically, streamline diffusion and crosswind diffusion were activated, as they are consistent stabilization methods that do not perturb the original transport equations. In contrast, isotropic diffusion, an inconsistent stabilization method that adds artificial diffusion, was disabled to preserve the accuracy of the concentration field.

To attain a concentration gradient, a three-inlet CGG was set up in the simulation. Its inlet conditions were defined as Inlet1 = 0 mol/L, Inlet2 = 1 mol/L, and Inlet3 = 0 mol/L. Simultaneously, the model assumed a no-slip boundary condition for all channel walls and set the static pressure at the channel outlet to 0 Pa as a reference. Seven Re values of 0.1, 0.5, 1, 10, 50, 100, and 200 were selected as the main parameters for the comparative experiments. At the microfluidic scale, in the low-Re regime (Re << 10), viscous forces dominated, the flow remained smooth, and the influence of geometric structures was relatively weak. When the Re was increased to the 50–200 range, inertial forces became significantly enhanced, leading to strong coupling with nonlinear effects such as secondary flows and flow separation induced by the stepped geometry. Setting the upper parameter limit at Re = 200 was therefore essential for systematically revealing the ultimate performance of the device and its unique flow–gradient coupling behavior under inertia-enhanced conditions.

## 4. Establishment of the Numerical Model for Concentration Gradients in the Three Devices

### 4.1. Velocity Field Visualization and Cross-Section Selection Method

**Figure 2:**
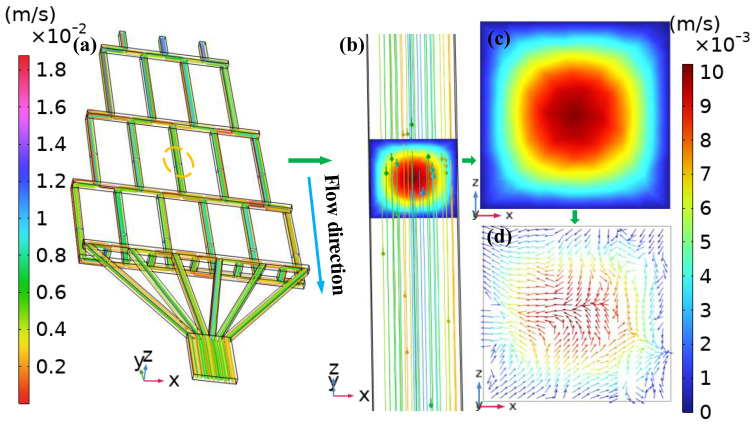
Variation in velocity gradients for DVS-GG at Re = 50, the blue arrow indicates the direction of fluid flow: (**a**) The overall velocity streamline plot of the microchannel. (**b**) The velocity streamline and cross-sectional plot of the middle channel at the third layer (Channel 3). (**c**) The cross-sectional velocity plot in the middle channel at *y* = −6 mm. (**d**) The cross-sectional velocity vector of the middle channel at *y* = −6 mm.

To illustrate the method for obtaining velocity vector plots at each layer, Figure 2 shows an example for DVS-GG at Re = 50. Figure 2a shows the overall velocity streamline plot of the microchannel at Re = 50. The left of the figure contains the overall velocity legend for the structure. The blue arrow indicates fluid flow along the negative *y*-axis direction. The yellow circle represents the selected locations for Figure 2b. Figure 2b shows the velocity streamline and cross-sectional plot at the third layer, *y* = −6 mm. This location selects the middle channel along the *x*-axis direction. The velocity legend on the right indicates that, due to fluid viscosity, the maximum velocity in the third layer was one order of magnitude smaller than the overall maximum flow velocity. Figure 2c is the *z*-*x* plane cross-sectional plot at this point. It was observed that the fluid velocity exhibited a clear gradient change across the section. The velocity was highest at the center of the microchannel and slower near the microchannel walls. Furthermore, the velocity change showed an iso-valued trend. This velocity difference successfully verified the typical characteristics of pressure-driven laminar Poiseuille flow. Figure 2d is the velocity vector plot on the *z*-*x* plane cross-section at *y* = −6 mm. The direction of the velocity arrow illustrates the changes in velocity forces. The figure clearly shows the compression of the arrows, which was prone to causing turbulent flow phenomena. This local acceleration played a crucial modulating role in the final mixing effect and gradient morphology.

### 4.2. Mesh Convergence Verification and Numerical Stabilization Strategies

**Figure 3:**
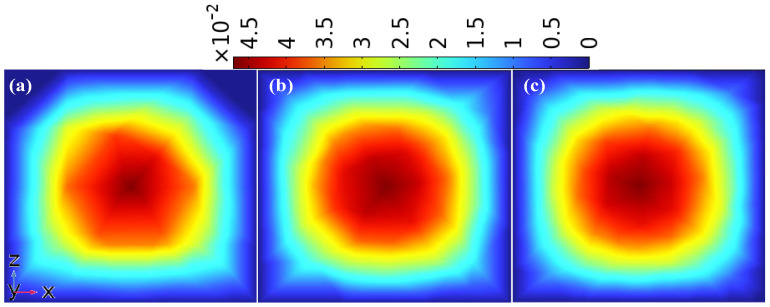
Velocity contours in the DVS-GG device under different mesh qualities at Re = 200: (**a**) 394,340 elements, (**b**) 1,319,079 elements, (**c**) 2,026,011 elements.

The computational domain was discretized using a structured free tetrahedral mesh. Mesh refinement was performed near the channel walls to accurately capture velocity gradients and concentration boundary layers. To investigate the influence of mesh quality on the characterization of fluid flow structures, the velocity contour plots obtained from three finite element meshes with different element numbers were compared for the mid-plane cross-section of the third channel layer (y = −6 mm) in the DVS-GG device under a high Reynolds number (Re = 200), as shown in Figure 3. The velocity contours in Figure 3a, b and c correspond to simulations performed with meshes consisting of approximately 394,340, 1,319,079 and 2,026,011 elements, respectively. The color legends were uniformly scaled in m/s. In all three meshes, the velocity distribution was observed to be highest in the central region and gradually decreased toward both sides. However, the mesh density was found to have a significant impact on the accuracy of velocity–gradient representation. In Figure 3a, the velocity contours exhibited step-like jumps and local irregularities, especially in the near-wall regions where the velocity gradient was steep. This was attributed to the relatively large element size, which could not adequately resolve the sharp variations within the boundary layer. With mesh refinement, the velocity contours in Figure 3b became more continuous, and the shape of the high-speed core region was captured more clearly. The transition of the velocity gradient from the center toward the sides is also better represented. In Figure 3c, the velocity gradient appears highly smooth and continuous. The extent and morphology of the high-speed core were accurately captured, and the transition from the near-wall low-velocity zone to the central region was very gradual, with no obvious numerical dispersion artifacts. These observations indicate that increasing the mesh resolution substantially enhances the ability to resolve gradients in the velocity field. Therefore, the fine mesh with approximately 2,026,011 elements was adopted for all subsequent simulations to ensure the reliable numerical reproduction of complex flow structures in the microchannel. A stationary solver was employed, in which the flow field was solved using the Laminar Flow interface, and the concentration field was solved using the Transport of Diluted Species interface, with a one-way coupling implemented between the two. To ensure the stability and reliability of the numerical solution at high Re, the inertial stabilization option was enabled in the solver, and a parametric stepwise sweep was adopted to progressively compute from lower to higher target Re.

**Figure 4:**
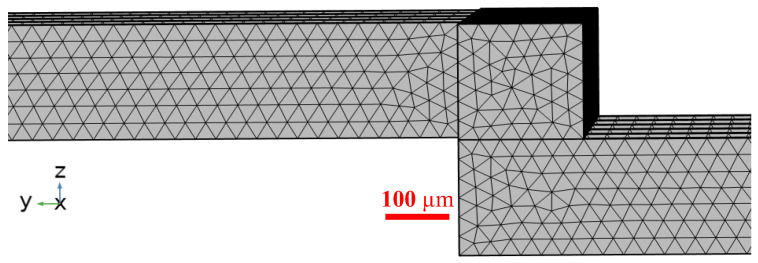
Mesh quality assessment in the transition region from the third to the fourth layer.

The structure of the mesh in the transition region (from the third to the fourth layer) was analyzed based on the provided screenshot and corresponding data, as shown in Figure 4. A scale bar of 100 μm was included to indicate the mesh size. The mesh consisted of approximately 2,026,011 elements, with a maximum aspect ratio below 5:1, indicating good overall mesh quality. The analysis indicated that the mesh in this region was characterized by well-structured features, with elements arranged in an orderly manner. High regularity was exhibited in both the main flow direction (opposite to the *y*-axis) and the cross-sectional plane. The mesh size transition from the third to the fourth layer was continuous and gradual, and no abrupt element contraction or distortion was observed. This demonstrated that the mesh gradient was properly controlled, which contributed to ensuring the stability of the numerical computations.

### 4.3. Analysis of Velocity Distribution Characteristics in Microchannels

**Figure 5:**
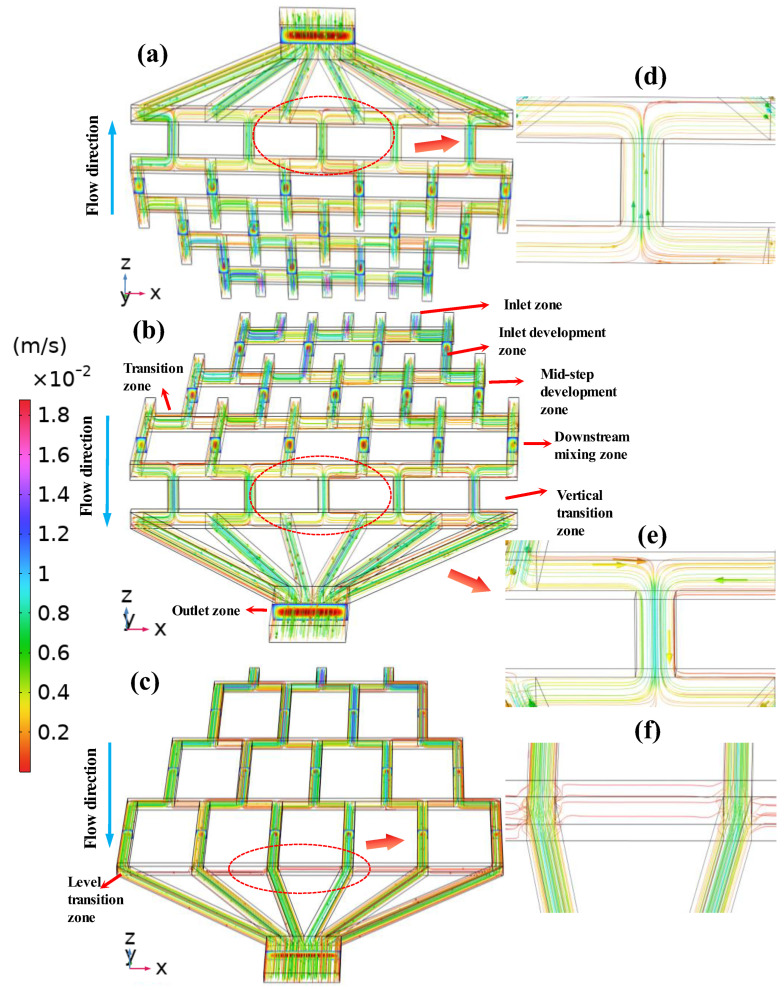
Streamline plots of the flow velocity along the main channel in the three devices (Re = 50): (**a**) DVS-GG device, (**b**) UVS-GG device, (**c**) SHC-GG device. (**d**) Magnified view of the vertical transition zone in the DVS-GG device. (**e**) Magnified view of the level transition zone in the UVS-GG device. (**f**) Magnified view of the level transition zone in the SHC-GG device.

To systematically analyze the performance of the CGG and elucidate the impact of its stepped geometry, the device structure was divided into six consecutive mixing zones along the flow direction: the inlet zone, the inlet development zone, the mid-step development zone, the downstream mixing zone, the transition zone, and the outlet zone, as shown in Figure 5. The transition zone located upstream of the outlet zone and extending along the *x*-direction of the channel exhibited a clear distinction between the SHC-GG and the UVS-GG/DVS-GG configurations, corresponding to the level transition zone and the vertical transition zone, respectively. Velocity and concentration fields were then examined at several representative cross-sections to capture key stages of flow development. The selected six consecutive mixing zones in the *z*-*x* planes corresponded to distinct morphological regions along the *y*-direction: the inlet development zone (*y* = −2 mm), the mid-step development zone (*y* = −6 mm), the downstream mixing zone (*y* = −10 mm), and the final outlet plane (*y* = −17 mm). The y-coordinates were chosen to be in the mid-region of each channel in order to acquire more representative flow and concentration information. These locations were chosen to capture the initiation, development, and stabilization of flow structures, particularly the secondary flows induced by vertical steps. Furthermore, to directly visualize the effect of step geometry, the overall streamline patterns of the three devices along the main channel were presented. Figure 5a, b and c show the comparison diagram of velocity streamlines among the DVS-GG (settling–lifting), UVS-GG (climbing–falling), and SHC-GG (straight–horizontal) devices at Re = 50, respectively. The color bar indicates the velocity range in the fluid streamline plot. The blue arrow indicates the direction of the fluid flow (along the negative *y*-axis) in the velocity streamline plot. As shown in Figure 5a and b, the velocity streamlines in the DVS-GG and UVS-GG devices, respectively, show how the flow followed the path of the main channel as it stepped upward and downward in elevation at the step zones prior to the outlet. A comparative analysis based on the velocity color bar showed that the flow velocity was significantly higher in the vertical transition zones of the DVS-GG and UVS-GG devices than in the level transition zone of the SHC-GG device. These three zones are detailed in Figure 5d, e and f, respectively, which are magnified views of the regions marked by red dashed lines in Figure 5a–c. This result confirmed that the stepped geometry induces distinct local flow acceleration. This acceleration enhanced fluid shear and stretching, effectively promoting flow disturbance and interface folding, thereby playing a crucial regulatory role in the final mixing efficiency and concentration gradient morphology.

Next, by comparing the velocity vector diagrams for each mixing zone within the DVS-GG structure using Re = 1 as an example, the evolution of non-uniformity in the flow structure across the channel cross-section and the propagation range of disturbances were revealed, and the changes in flow characteristics from the inlet to the outlet were analyzed. To simplify the analysis, the middle channel velocity vector plots on both sides were extracted from the symmetrical structures of each layer for comparison.

**Figure 6:**
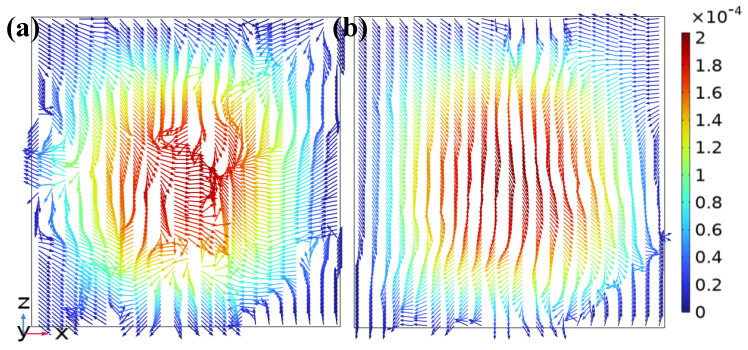
Velocity vector distributions on the *z*-*x* cross-sections at the inlet development zone in the DVS-GG device (Re = 1): (**a**) Channel 2, (**b**) Channel 3.

At the inlet development zone (*y* = −2 mm), the flow had just entered the structure and was not yet fully developed, with velocities close to the initial values. In cross-sections, the velocity at the center was significantly higher than at the sides, as shown in Figure 6a,b. The vector directions were primarily along the positive *x*-axis but exhibited slight disturbances. The color gradient showed the highest velocity in the central region, decreasing outward to low-speed zones, with a clear gradient change. This stage was the flow establishment period, dominated by the main channel. Side channels were significantly affected by wall friction, resulting in a larger low-velocity region, and strong vortices had not yet formed.

**Figure 7:**
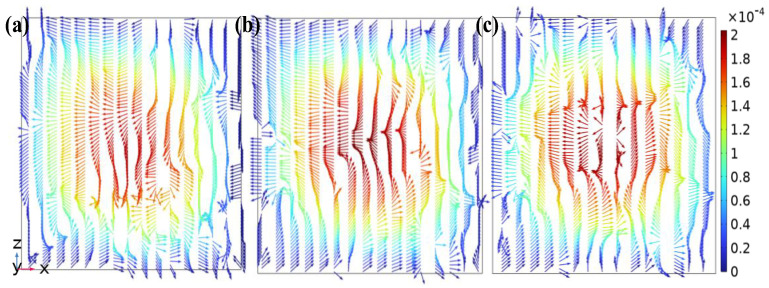
Velocity vector distributions on the *z*-*x* cross-sections at the downstream mixing zone in the DVS-GG device (Re = 1): (**a**) Channel 2, (**b**) Channel 3, (**c**) Channel 4.

Reaching the mid-step development zone (*y* = −6 mm), the flow gradually stabilized but still exhibited pronounced non-uniformity, as shown in Figure 7. The low-velocity zone in this region was reduced in extent compared to that in the inlet development zone. In Figure 7b, the velocity arrows are noticeably stretched in the *x*-axis direction by bidirectional forces. Furthermore, in Figure 7c, the high-velocity region develops a stronger velocity component in the *z*-axis direction, approximating the nascent form of a vortex. In Figure 7a, the primary flow direction is along the negative x-axis, indicating a stable, well-developed flow state.

**Figure 8:**
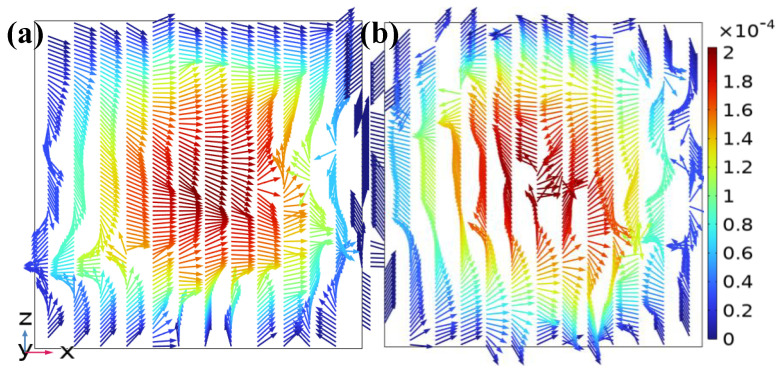
Velocity vector distributions on the *z*-*x* cross-sections at the downstream transition zone in the DVS-GG device (Re = 1): (**a**) Channel 3, (**b**) Channel 4.

At the fourth layer, the downstream transition zone (*y* = −10 mm), the high-speed areas in the cross-sections of Figure 8a,b are larger than those in the two aforementioned regions. The compression of opposing velocity arrows became more evident, and the vortex structure became clearer. Figure 8b shows multiple small-scale vortices distributed around the channel center. The velocity vectors exhibited approximate “ring-like” or “spiral” patterns. The area enclosed by low-velocity regions was significantly reduced. The result of the stepped structure inducing complex flow patterns to enhance mixing efficiency became more prominent.

**Figure 9:**
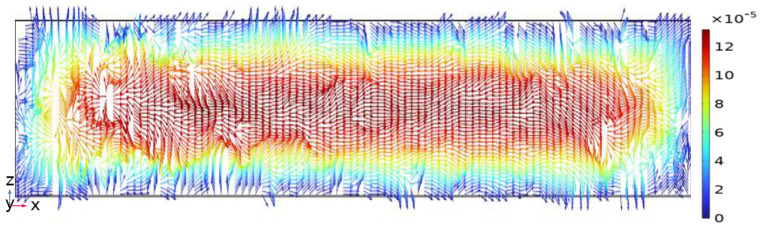
Velocity vector distribution on the *z*-*x* cross-section at the outlet zone in the DVS-GG device (Re = 1).

At the outlet zone (*y* = −17 mm), after passing through the previous split channels, the fluid converged into one main channel, as shown in Figure 9. The channel suddenly widened and the overall velocity decreased and tended to level off, but it still maintained certain fluctuations in the *x*-axis direction. The high-velocity zone formed a continuous band spanning the entire length of the channel cross-section. The area of medium-to-low velocity layers in the edge regions decreased, while very-low-velocity zones constituted a “boundary layer”. The color distribution showed a strong contrast between the center and edges. As the outlet layer microchannel was wider than other structures, multiple instances of streamline convergence occurred, which could induce fluid vortices. The structure provided continuous disturbance, ensuring a certain degree of fluid mixing capability.

**Figure 10:**
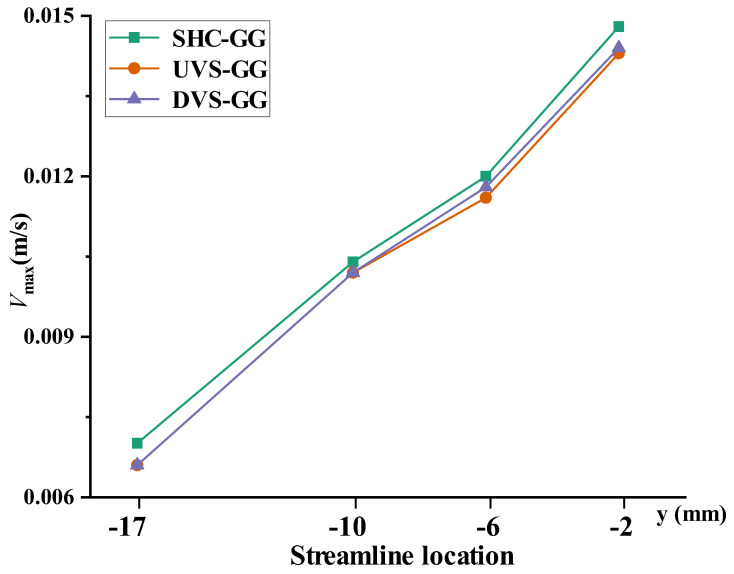
Maximum velocity values at the microchannel cross-sections of each layer for the three devices.

Based on the provided velocity distribution plots, the relationship of maximum velocities across the four layers within the main channels of the three devices—UVS-GG, DVS-GG, and SHC-GG—can be compared, as shown in Figure 10. The data in the figure indicate that the *x*-axis represented the four *z*-*x* cross-sectional velocity vector plots along the fluid flow direction from *y* = −17 mm to *y* = −2 mm. The *y*-axis represented the maximum velocity values measured at the four cross-sections within each layer for the three respective devices. From the figure, it was observed that for all three devices, the velocity was lowest at the position closest to the outlet. From the inlet to the outlet, the maximum velocity values showed a decreasing trend layer by layer. For the cross-sections taken at *y* = −2 mm and *y* = −6 mm along the flow direction, the velocities of the three devices exhibited a decreasing relationship, with SHC-GG having the highest fluid flow velocity and UVS-GG the lowest. However, for the cross-sections taken at *y* = −10 mm and *y* = −17 mm, the maximum velocities of the UVS-GG and DVS-GG devices were similar, while the SHC-GG device still maintained the highest values. The differences in the velocity distribution plots visually confirmed that the three geometric configurations enable control over the internal flow field structure by altering the flow path and gravitational potential energy. This provides a crucial basis for further regulating the convective transport processes during concentration gradient formation.

### 4.4. Analysis of Pressure Drop Variation with Reynolds Number in Microchannels

**Figure 11:**
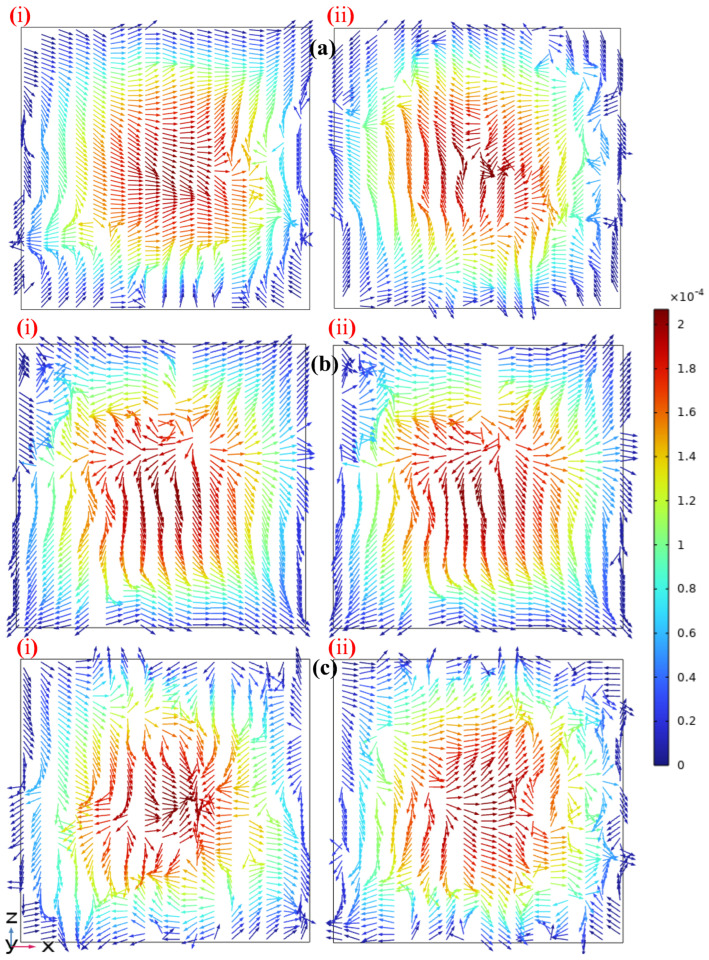
Comparison of velocity vector diagrams in the middle channels of the fourth layer for the three devices (Re = 1): (**a**) UVS-GG device, (**b**) DVS-GG device, (**c**) SHC-GG device, (i) Channel 3, (ii) Channel 4.

To compare the fluid flow characteristics induced by the three types of devices within microchannels of fixed geometric parameters, Figure 11 shows the velocity vector field at the cross-section *y* = −10 mm for Re = 1. This structural layer contained six microchannels. To focus on core flow features and simplify the analysis, the two symmetric middle channels, Channel 3 and Channel 4, were selected for detailed study as they represent the overall flow state in this layer. The color map in the legend on the right visually illustrates the velocity differences under different structures.

Figure 11a shows the velocity vector plot of the UVS-GG structure at the downstream transition layer (*y* = −10 mm), revealing unique flow characteristics. This structure broke the symmetrical flow state of the fluid. At a low Re, where viscous forces dominate the fluid flow, the streamlines in cross-section Figure 11(ai) are uniformly stretched toward the right wall of the microchannel. In cross-section Figure 11(aii), the fluid exhibits asymmetric flow within the channel with opposing flow directions and a locally compressed “spiral” tendency. This feature induces complex changes in the flow field.

Figure 11b, the velocity vector plot at the same location for the DVS-GG structure, displays flow characteristics different from those in Figure 11a. The streamline flow structures in Figure 11(bi) and Figure 11(bii) are similar, with flow tending toward both ends of the microchannel sidewalls, forming an asymmetric flow pattern. The direction and distribution of shear forces in the high-speed regions are evident, showing a locally compressed spiral tendency that was prone to increasing mixing efficiency.

Figure 11c shows the velocity vector plot for the SHC-GG structure. Compared to the other two devices, its flow state was more complex. Influenced by shear forces, the velocity arrows converged noticeably toward each other, which was likely to induce a tendency toward fluid turbulence and enhance mixing.

**Figure 12:**
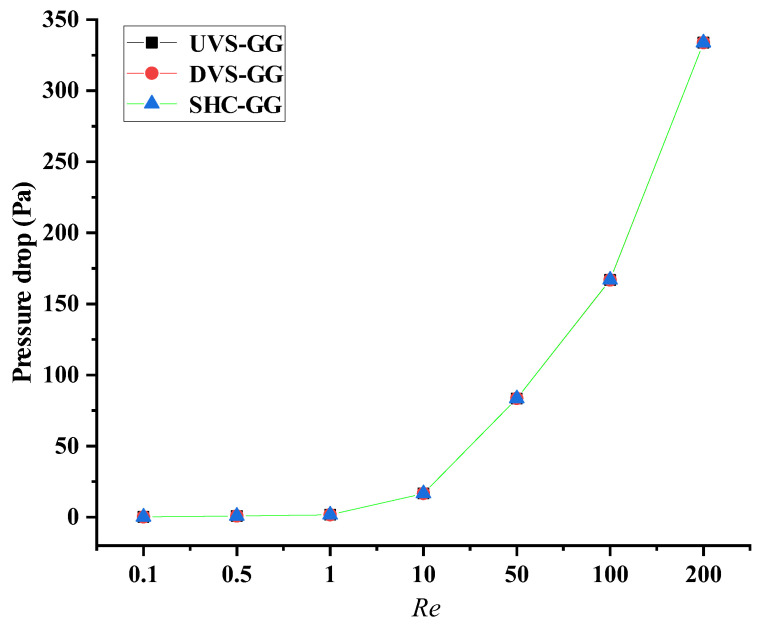
Physical relationship between pressure drop and Re for the three devices.

The data in Figure 12 clearly illustrate the physical relationship between the pressure drop and Re for the three types of devices. In this study, the pressure drop represented the pressure difference between the inlet and the outlet. The *x*-axis represented the seven Re parameters ranging from 0.1 to 200, while the *y*-axis represented the pressure drop values. As can be seen from the figure, the pressure drop values for the three devices were similar at identical parameters. Furthermore, the pressure drop increased with increasing Re. In pressure-driven flow, the driving pressure increased with higher flow velocities to overcome both the viscous resistance and the inertial resistance of the fluid. Given that the Reynolds number was proportional to the flow velocity, this phenomenon reflected a universal law in fluid dynamics.

In the high-Re region (Re ≥ 50), the curve descended steeply with a relatively large negative slope, indicating that the pressure drop rose rapidly as the Re increased. This likely corresponded to a flow state dominated by inertial forces, where the flow was transitioning from laminar to turbulent, and the resistance mechanism underwent significant changes. In the medium-Re range (Re ≈ 1–10), the curve tended to flatten, with a slope approaching zero, indicating that the variation in pressure drop with Re weakened and stabilizes. In the low-Re region (Re ≤ 1), the curve was almost horizontal, with a slope tending toward zero. This reflected that the pressure drop essentially no longer changed with the Re number, and the flow entered a stable regime dominated by viscous forces.

While the three devices exhibited similar overall pressure drops at identical Re, this observation does not contradict their significantly different internal flow structures and mixing behaviors. The overall pressure drop is predominantly governed by the macroscopic geometric features that determine frictional resistance along the flow path and changes in kinetic energy, including the inlet/outlet cross-sectional areas and the main channel length, which were comparable across all designs [29,30]. Although the local perturbations introduced by the stepped geometries dramatically alter the flow patterns, the additional form drag they generate constitutes only a small fraction of the total pressure drop. The essential role of the stepped geometry lies in breaking the flow symmetry and converting part of the streamline kinetic energy into transverse secondary flows and vortex energy [31,32]. This spatial redistribution of energy effectively enhances fluid stretching and folding, thereby substantially improving mixing efficiency and promoting the formation of complex concentration gradients without significantly increasing the overall energy dissipation of the system. Consequently, the result of similar pressure drops, yet distinct performance, highlights the efficiency of the stepped design, which enables precise control over the mixing and gradient-formation processes through the active redistribution of internal flow energy without a noticeable increase in driving power consumption.

## 5. Analysis of the Concentration Gradient Generator Model

### 5.1. Formation and Evolution of Concentration Gradient Contours

**Figure 13:**
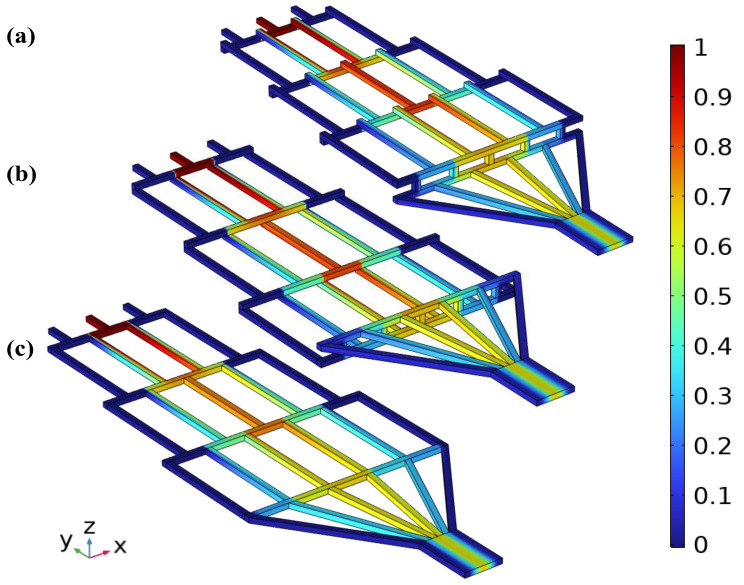
Simulated concentration contours of the three types of devices at Re = 10: (**a**) UVS-GG device; (**b**) DVS-GG device; (**c**) SHC-GG device.

In this set of simulations, with the fluid properties and channel geometry held constant, the Re is proportional to the flow velocity. In microchannels of equal length, lower flow velocities resulted in higher mixing efficiency. The concentration gradient profiles of the three devices at Re = 10 are shown in Figure 13. (Note: Based on the context, this refers to the concentration contour plots, not Figure 13, which shows the pressure drop. There might be a figure numbering inconsistency.) This was a set of comparative 3D simulation results, where the color legend on the right indicated the specific solute concentration distribution patterns.

Figure 13a, b, and c represent the UVS-GG, DVS-GG, and SHC-GG devices, respectively. From the three sets of images, it could be observed that, as the fluid flows layer by layer towards the outlet, mixing gradually occurred in the central part of the microchannels. The mixing effect became more pronounced closer to the outlet. In contrast, the mixing effect of the fluid on both symmetrical sides of the structure was extremely low, while the mixing was most effective in the central structure, proceeding stepwise by layer. It was precisely this fractal principle that enabled the CGG to create the required gradient environment at the outlet.

Comparing the three profiles, the overall shapes of the UVS-GG and DVS-GG devices were very similar. For the SHC-GG device, the red color intensity clearly decreased starting from the third layer, indicating that the local mixed concentration here was around 0.7 mol/L. In contrast, the UVS-GG and DVS-GG devices maintained concentrations around 0.9 mol/L at the same stage. In the fourth layer, the mixed area on both sides of the central functional channel in the SHC-GG device increased compared to the other two devices. Based on Equation (3), the overall comparison revealed that the UVS-GG device exhibited better mixing performance, with a mixing efficiency value of 0.545. However, the DVS-GG device yielded a very close result, with a negligible difference of approximately 0.0001.

**Figure 14:**
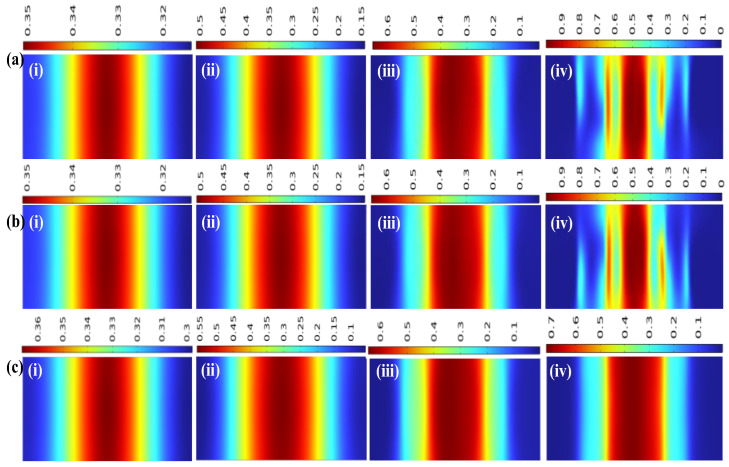
Concentration cross-sectional plots at the outlet of the three devices: (**a**) UVS-GG device, (**b**) DVS-GG device, (**c**) SHC-GG device, (i) Re = 0.1, (ii) Re = 1, (iii) Re = 10, (iv) Re = 200.

To better observe the concentration gradient at the outlet, *z*-*x* cross-sectional concentration plots at *y* = −17 were selected for Re = 0.1, 1, 10, and 200, corresponding to Figure 14(i), (ii), (iii), and (iv), respectively. Figure 14a, b, and c represent the UVS-GG, DVS-GG, and SHC-GG devices, respectively, with the legend at the top of each plot corresponding to its respective figure.

Observation of the overall mixing revealed a consistent trend across all device types: as the Re increased, the maximum concentration value in the cross-sectional plots rose correspondingly. Conversely, the minimum concentration value exhibited an inverse relationship, decreasing as the Re increased. This illustrated the state of fluid mixing as a function of the Re. Examining the plots for the three devices, at low to medium Re values, the concentration showed a symmetrical and smooth decrease from the high concentration in the center of the microchannel to the lower concentrations at both sides. The legends and graphical changes for UVS-GG and DVS-GG were completely consistent. In contrast, for the SHC-GG at the same Re, the maximum and minimum concentration values were respectively higher and lower compared to the other two types.

For the UVS-GG and DVS-GG devices at high Re values, the fluid concentration exhibited fluctuating changes. The cross-section showed an approximate oscillatory wave-like variation from high to low concentrations. In contrast, the SHC-GG device maintained a very smooth profile consistent across both high- and low-Re conditions.

### 5.2. Analysis of the Morphological Characteristics of the Concentration Gradient Curves

**Figure 15:**
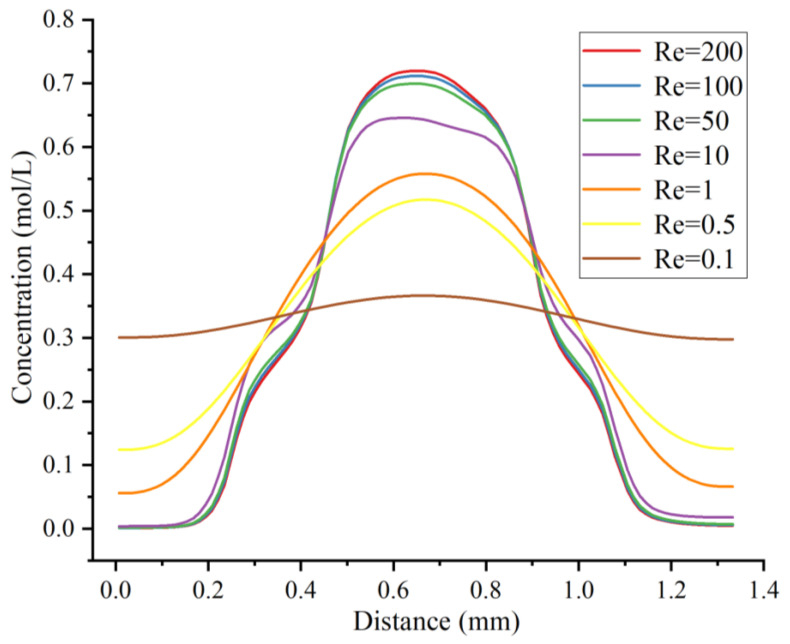
Concentration gradient curve for the SHC-GG device.

The concentration gradient curves generated along the centerline of the outlet cross-section (*y* = −17 mm) of the SHC-GG device for seven different Reynolds numbers are shown in Figure 15. Since the fluids injected from the two side inlets had a concentration of 0 mol/L and that from the central inlet injected was 1 mol/L, the curve exhibited a Gaussian distribution. At Re = 0.1, the mixing effect was optimal, with the concentration distribution tending toward uniformity. The concentration curve was gentle, the concentration gradient was minimal, the peak concentration was 0.35 mol/L, and the concentration gradient difference was only 0.05 mol/L. When the Re ranged from 0.5 to 1, the concentration curve remained monotonic, with the peak concentration around 0.5 mol/L and the lowest concentration around 0.1 mol/L.

As the Re increased from 10 to 200, the concentration gradient became more pronounced. The ends of the concentration curve remained at 0 mol/L, and the curve began to exhibit fluctuations. The peak concentrations for the Re from 50 to 200 were very close, around 0.7 mol/L. The symmetrical concentration profiles on both sides of the peak were very similar. Furthermore, the larger the Re, the lower the mixed concentrations on both sides of the peak. Overall observation indicated that the peak concentration was inversely proportional to the increase in the Re, while the symmetrical concentrations on both sides of the peak were directly proportional to the increase in the Re, and symmetrical fluctuations appeared on both sides of the peak.

**Figure 16:**
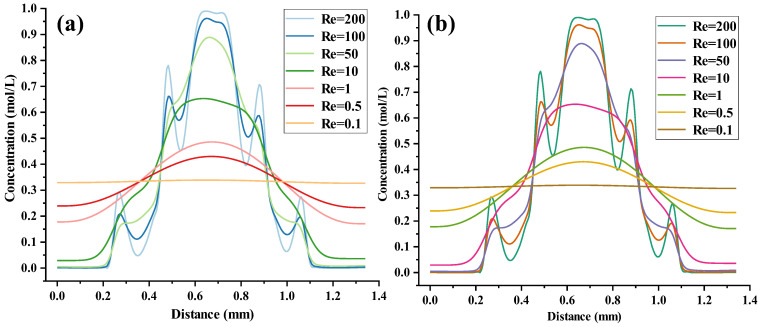
Concentration gradient distribution curve for two devices: (**a**) UVS-GG devices, (**b**) DVS-GG device.

The concentration gradient distribution curves for the UVS-GG and DVS-GG devices are shown in Figure 16a and b, respectively. The curves were also generated along the center line of the outlet cross-section (*y* = −17 mm) of the two devices at seven Re values. The Re remained the seven numbers ranging from 0.1 to 200. The parameter values of the UVS-GG device were approximate with those for the DVS-GG device. The numerical comparison results demonstrated that the concentration gradient distributions of the two devices are generally consistent. The main difference lay in the region to the right of Re = 200, where a maximum relative difference of approximately 19% was observed; as the Re decreased, the difference diminished significantly, and at Re = 0.5, the data from the two devices became completely consistent. When the Re was 0.5 and 1, the concentration gradient curves were monotonic. The corresponding maximum peak concentrations were 0.4 mol/L and 0.45 mol/L, while the minimum concentration values were 0.17 mol/L and 0.24 mol/L, respectively, indicating a higher mixing efficiency than that of the SHC-GG device. At Re = 10, the concentration gradient curve became non-monotonic, with the minimum concentration at both ends being 0.03 mol/L. For a Re of 200 and 100, the highest peak values were both greater than 0.95 mol/L. The concentration gradient curves exhibited five fluctuating peaks, with each peak value proportional to the magnitude of the Re. After the peak transitions to the intermediate position near the minimum concentration, the concentration became inversely proportional to the magnitude of the Re. At Re = 50, the concentration gradient peak was 0.87 mol/L. Compared to the SHC-GG device, the UVS-GG device showed higher mixing efficiency when the Re ≤ 1, but lower mixing efficiency when the Re ≥ 10. Despite the opposing step orientations, the UVS-GG and DVS-GG devices yielded nearly indistinguishable outlet concentration profiles, including peak locations, under the same Re conditions. This similarity suggested that the absolute direction of the vertical step might be less critical for the final macroscopic gradient pattern than the presence of the step itself, which introduced a periodic disturbance that coupled with inertial forces.

Based on the aforementioned data, Table 1 presents the mixing efficiency (ME) values calculated from Equations 5 and 6, which are reported to five decimal places. Using these data, the performance of the three devices was systematically compared across different Re. The SHC-GG device was observed to exhibit the lowest mixing efficiency under all tested Re conditions, indicating relatively poor mixing performance. When the Re was 10 or higher, the mixing efficiency of the UVS-GG device was found to be slightly higher than that of the DVS-GG device, although the difference between them remained below 0.0045. In the low-Re regime where the Re was 1 or less, the efficiency values of the two stepped-structure devices were nearly identical. These results clearly demonstrated that, in terms of mixing efficiency, the layered vertical-step configurations of the UVS-GG and DVS-GG were superior to the straight horizontal-channel design of the SHC-GG.

This phenomenon, where mixing efficiency at low Reynolds numbers was superior to that at high Re, was closely related to the residence time of the fluid within the microchannel. For a fixed channel length, the residence time was inversely proportional to the flow velocity. Therefore, a low flow velocity, corresponding to a low Re, resulted in a longer residence time, which provided sufficient opportunity for molecular diffusion to homogenize the concentration field, thereby yielding higher mixing efficiency. Conversely, a high flow velocity, corresponding to a high Re, significantly shortened the residence time. Even though the stepped geometry might have induced secondary flows at high Re, the limited residence time restricted the extent of molecular diffusion, leading to lower mixing efficiency. This observation was consistent with the findings of Hossain et al., who reported that in passive micromixers with fixed channel lengths, mixing performance in the diffusion-dominated regime was indeed governed by residence time, with lower flow rates promoting more complete mixing due to prolonged diffusive transport [33].

### 5.3. Characteristic Analysis and Mechanism Investigation of Multi-Peak Oscillatory Concentration Gradients

**Figure 17:**
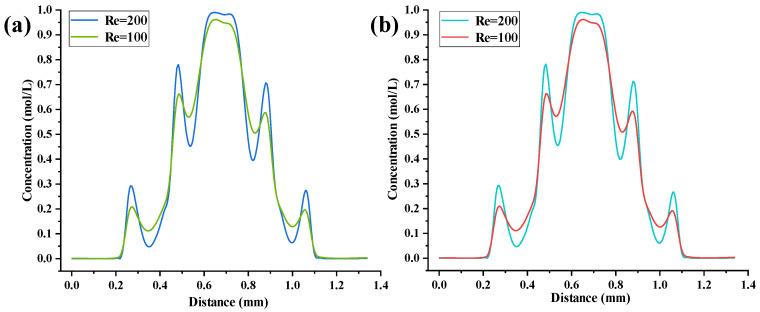
Multi-peak oscillatory concentration gradients for two devices: (**a**) UVS-GG device, (**b**) DVS-GG device.

During the forced convective mixing process within the vertical main channels, the mixed fluid exhibited characteristics of non-uniformity and dynamic instability at the microchannel outlet. As shown in Figure 17a,b, the concentration distribution curves along the *x*-coordinate from 0 to 1.4 mm for both the UVS-GG and DVS-GG devices under Re = 100 and Re = 200 did not exhibit a monotonic decay trend. Instead, they displayed alternating periodic peaks and troughs, exhibiting a multi-peak oscillatory concentration gradient distribution pattern. Comparing the two devices, the numerical values of the multi-peak oscillations were almost identical, with differences appearing only at the right end near *x* = 0.65 mm. The concentration curve successively showed five distinct peaks near approximately *x* = 0.26 mm, 0.48 mm, 0.65 mm, 0.88 mm and 1.05 mm, where the amplitude was most significant. This indicated that at higher Re, enhanced flow inertia could induce more intense vortex shedding or secondary flow structures, thereby promoting repeated local aggregation and dissipation of the mixed substances.

This multi-peak oscillation phenomenon could be attributed to the evolution of periodic flow structures induced by the coupling between inertial forces and the stepped geometry, a mechanism well-established for generating complex flow patterns in structured microchannels [27,32]. Fluid parcels underwent continuous stretching, folding, and reorganization, leading to an oscillatory evolution of the interfacial concentration gradient. Furthermore, since the Re was proportional to flow velocity, its increase amplified flow instability, causing an increase in the amplitude and potentially the frequency of concentration oscillations. The inversion of the channel geometry altered the balance between transverse pressure gradients and centrifugal forces, thereby modulating the mode and spatial distribution of the oscillations. The multi-peak concentration distribution indicated that the fluid was not uniformly mixed across the outlet cross-section. Instead, there were multiple alternating regions of high and low local concentrations. This phenomenon was caused by geometry-induced secondary flows, vortices, or shear layering, which led to solute accumulation in specific regions, forming concentration “peaks”.

While the UVS-GG and DVS-GG configurations yielded similar outlet concentration profiles, the direction of vertical stepping was found to distinctly alter the internal flow evolution by modifying local pressure gradients and centrifugal balances. This directional difference influenced the formation location and intensity of secondary vortices, thereby providing differentiated initial disturbances to the flow. In comparison with the three conventional types of multilayer CGGS, which operated on passive splitting and diffusion at low Re, the present stepped design actively coupled flow inertia with periodic geometric variations. This coupling enabled the dynamic programming of gradient morphology across a range of Re, supported the generation of high-spatial-frequency multi-peak concentration patterns, and offered enhanced capabilities for applications demanding rapid mixing, inertial particle manipulation, or spatially patterned biochemistry environments. Notably, based on the successfully revealed biological mechanism whereby the nonlinear dynamic equilibrium of the MinD/E oscillatory gradient enables plasticity-driven regulation of cell division, this natural paradigm of oscillatory concentration gradients demonstrates broad application potential, offering important bio-inspiration for the artificial design and programming of complex biochemical microenvironments [34].

For three-dimensional structures with vertical steps such as the UVS-GG and DVS-GG, multilayer soft lithography can be employed using PDMS along with SU-8 photoresist molds and plasma bonding for fabrication. This technique is well established and enables precise control over channel dimensions and inter-layer alignment. An alternative rapid-prototyping approach is high-resolution projection micro-stereolithography 3D printing, whose accuracy is sufficient to reproduce the stepped features described in this work. In addition, laser-cutting of thermoplastic sheets such as PMMA combined with a thermal bonding process offers a low-cost and fast route for fabricating such three-dimensional architectures. To reach the flow rates required for high-Re conditions, high-pressure syringe pumps or precision pressure controllers can be used. For SHC-GG operation at a low Re, conventional syringe pumps are adequate to deliver sufficiently stable and smooth flow. During implementation, it should be noted that the elevated pressures associated with high Re may pose a challenge to the bonding strength of the chip. Furthermore, the fabrication accuracy of the step corners could influence vortex generation, which is a key point to consider when comparing experimental results with simulations.

## 6. Conclusions

Through a numerical simulation and systematic comparison of three types of CGGs—the UVS-GG, DVS-GG, and SHC-GG—this study clarified the core regulatory mechanisms of geometric design and flow parameters on concentration field formation and mixing efficiency in microfluidic devices. At low Re, where viscous forces dominate, concentration gradients were primarily formed by molecular diffusion. As the Reynolds number increased to the medium range (Re = 10–50), inertial effects became significantly enhanced. The inclined and declined stepped geometries induced local secondary flows and velocity non-uniformities through periodic directional changes, thereby disrupting the diffusion-dominated transport balance. Particularly at Re = 100 and 200, the resonant coupling between the stepped geometry and inertial forces gave rise to a spatially periodic “multi-peak oscillatory concentration gradient.” This phenomenon was a direct manifestation of internally ordered secondary flow structures, demonstrating that complex programming of concentration distributions was obtained through geometric design. Pressure drop analysis indicated that, while the three devices exhibited similar pressure drops at identical Reynolds numbers, the stepped designs delivered enhanced mixing and gradient programmability without a proportional rise in driving pressure, thereby offering an energy-efficient alternative to conventional methods that rely on channel elongation or cross-section constriction. This study provides clear guidance for the rational design of microfluidic CGGs. If the goal is to generate monotonic and predictable gradients, the SHC-GG is suggested to be the optimal choice when operated at low to medium Re. For fundamental biological studies such as cell chemotaxis assays, where stable gradients are required, conventional CGGs operating at low Re have been proven effective. Conversely, if high-throughput mixing, rapid homogenization, or inertia-based particle sorting is desired, the UVS-GG or DVS-GG is suggested to be the optimal choice when operated at higher Re to leverage their convection-enhancement mechanisms. The discovered “multi-peak oscillatory concentration gradient” offers a new approach for creating complex biochemistry environments with spatial frequencies and patterns at the micro-scale. For high-throughput screening applications that demand rapid mixing and parallel testing of concentration conditions, integrated gradient generators are key components [35]. Furthermore, the ability to program spatial concentration patterns suggests potential in guiding the synthesis of materials with periodic structures, analogous to the use of oscillatory flow in polymer particle synthesis [36].

This study provides clear operational guidelines for the design of concentration gradient generators for different applications. In contrast to conventional tree-shaped CGGs based on flow-splitting or diffusion principles, this study uses numerical simulations to tune the gradient morphology by adjusting the flow velocity in the stepped structure using numerical simulations, enabling transitions from monotonic distributions to complex multi-peak patterns. The structurally simple SHC-GG produces monotonic gradients at Re ≤ 1, with its low shear stress under 1 Pa ensuring cell viability, making it highly suitable for fundamental biological experiments such as cell chemotaxis assays. In contrast, the UVS-GG and DVS-GG leverage inertia–geometry coupling to show rapid mixing or programmable complex concentration distributions at moderate to high Reynolds numbers ranging from 10 to 200. This capability gives them significant advantages in cutting-edge applications including high-throughput screening, inertial particle manipulation, and spatially patterned culture, and they can also be effectively used to guide cell arrangement or synthesize periodically structured materials. For cell-related studies, operation is recommended within Reynolds numbers of 10 to 50 to control shear stress, while non-biological applications can utilize higher Reynolds numbers up to 200 for optimal performance, which requires pairing with high-pressure driving systems. This advancement holds promise for promoting developments in single-chip multi-condition biological screening, patterned cell growth, and the synthesis of materials with periodic structures. Future work could further explore three-dimensional stepped configurations, multiphase flow systems, and active feedback control, driving the evolution of CGGs from simulation tools toward dynamically programmable manufacturing and synthesis platforms.

## Figures and Tables

**Table 1:** The mixing efficiency (ME) of three devices at different Re.

Device Type	ME at Re = 200	ME at Re = 100	ME at Re = 50	ME at Re = 10	ME at Re = 1	ME at Re = 0.5	ME at Re = 0.1
UVS-GG	0.49210	0.52227	0.54288	0.71472	0.93719	0.97602	0.99992
DVS-GG	0.48909	0.52094	0.53856	0.71459	0.93721	0.97602	0.99992
SHC-GG	0.63683	0.64508	0.65701	0.71056	0.84170	0.90468	0.99734

## Data Availability

The data are available from the corresponding author upon reasonable request.
